# Postoperative Acute Kidney Injury After Transcatheter Aortic Valve Replacement

**DOI:** 10.1007/s40140-024-00626-z

**Published:** 2024-04-01

**Authors:** Sebastian Ayala, Zhengmin Ma, Ke Peng, Fuhai Ji, David Li

**Affiliations:** 1 Department of Anesthesiology and Pain Medicine, University of California Davis Health, Sacramento, CA, USA; 2 The First Affiliated Hospital of Soochow University, Suzhou, People’s Republic of China

**Keywords:** TAVR, Acute kidney injury, Cardiac

## Abstract

**Purpose of Review:**

The purpose of this review serves to briefly summarize the current literature surveying the incidence of posttranscatheter aortic valve replacement acute kidney injury (TAVR AKI). Furthermore, this review extends itself to evaluate and potentially address modifiable risk factors, while acknowledging non-modifiable risk factors in the perioperative setting. These modifiable risk factors include but are not limited to access method, perioperative hypotension events, and need for blood transfusion in the setting of preoperative anemia.

**Recent Findings:**

Recent retrospective studies have highlighted the incidence of post-TAVR AKI, citing as high as 1 in 6 patients. Despite exclusion of patients with end-stage renal disease (ESRD) from pivotal TAVR trials, data shows that over 50% of high-risk patients suffer > 3a chronic kidney disease (CKD) and about 10% of them suffer > 4 CKD, with the risk of AKI increasing significantly at each stage of CKD advancement. Meta-analyses have shown that patients who underwent TAVR via transfemoral (TF) approach compared to those who had transapical (TA) or transaortic (TaO) approach had significantly less AKI post-TAVR. Furthermore, in patients who developed post-TAVR AKI, 55% of them had received *packed red blood cell* (pRBC) transfusion, while only 21% of the patients who did not receive pRBCs develop post-TAVR AKI (*p* = .002). Post-TAVR AKI has been shown in multiple studies that it is an independent risk factor for increased short term and long-term mortality. These findings together highlight both the clinical significance and continued perioperative vigilance to further improve clinical outcomes.

**Summary:**

This review aims to summarize recent literature regarding the association of AKI in the perioperative setting of TAVR. In addition, it parses the risk factors into both modifiable and non-modifiable risk factors. Furthermore, it provides some recommendations including procedure approach, appreciating transfusion implication, and most importantly, preventing hypotension events in the perioperative period.

## Introduction

Transcatheter aortic valve replacement (TAVR) has quickly become one of the common structural heart procedures performed in the last decades. TAVR has become even more popularized since the advent of PARTNER3 Trial in 2019. The multicenter, randomized study concluded that for patients with severe aortic stenosis who were at low surgical risk, the rate of the composite of death, stroke, or rehospitalization at 1 year was significantly lower with TAVR than with surgical aortic valve replacement (SAVR) [1]. While TAVRs have significantly improved morbidity and mortality in patients compared to SAVR [2], TAVR is not without complications. Acute kidney injury (AKI) after TAVR is not uncommon and is associated with increased morbidity and mortality. This literature review aims to summarize the current knowledge on AKI after TAVR and the associated risk factors, incidence, pathogenesis, and perioperative management strategies. AKI in this literature review for the intent of standardization is defined per VARC criteria, unless otherwise mentioned [3]. Briefly, this criterion utilizes the current definition of AKI which has been adopted by the majority of the nephrology community, including the KDIGO (Kidney Disease: Improving Global Outcomes) initiative [4].

## Incidence

Post-TAVR AKI is unfortunately a common complication, occurring in 1 out of 6 patients undergoing TAVR [5•]. It is also a complication that often lends itself to higher rates of overall mortality comparatively to non-AKI patients (15.9% vs. 0.8%; *p* < 0.01^5^). In addition, the length of stay is prolonged as well as the associated costs with the visit because of post-TAVR AKI. The incidence of post-TAVR AKI has varying degrees of reporting. The published rates of post-TAVR AKI typically vary from 10 to 30% depending on AKI criteria and/or definition as well as cited literature [6]. Furthermore, patients selected for TAVR often tend to be older and have a higher prevalence of preexisting chronic kidney disease (CKD), compared with SAVR cases [7]. Post-TAVR AKI is further risk stratified as the procedure often requires contrast administration. Other factors include the disruption of existing atherosclerotic plaque that is being manipulated during instrumentation of the procedure, producing increased risk and likelihood of both atheroembolic events, another well-known risk factor for post-TAVR AKI.

## Pathogenesis

The pathophysiology for post-TAVR AKI patients is most likely multifactorial as there are multiple etiologies that are to be considered when evaluating the pathophysiology driving AKI in this setting. For example, age is an important factor to consider given that most patients who are receiving TAVRs tend to be older. By the age of 70, the kidneys have lost between 30 and 50% of their cortical glomeruli due to ischemic changes, and of the remaining glomeruli, most have some degree of sclerosis, as well as concomitant tubular and vascular changes leading to functional changes including reduction of renal blood flow of up to 50% from the age of 20 to 80 [8, 9]. To exemplify this point, patients who have existing CKD have an exponentially higher odds ratio of developing AKI. For patients with CKD 4–5 (eGFR < 30 ml/min/1.73 m^2^) and CKD 3b-5 (eGFR < 45 ml/min/1.73 m^2^), the odds ratio of developing AKI was 2.9 (CI 2.4–3.7) and 1.45 (CI: 1.28–1.7), respectively. This interplay is clinically significant because the hazard ratio for all cause death for this same cohort 1 year later was 2.0 (1.38–2.89) and 1.44 (1.04–1.98), respectively [1]. AKI is not without predisposing risk factors. While measures are performed in the preoperative risk assessment including AKI in the calculation for the *European* System for Cardiac Operative Risk Evaluation (*EuroSCORE*) I/II or the Society of Thoracic Surgeons (STS) Score. To parse these risks, the risk factors will be divided into pre/peri/postprocedural categories and further stratify into modifiable vs. non-modifiable risk factors. This review aims to summarize post-TAVR AKI incidence, modifiable risk factors, and considerations that have been published in the literature in recent years (2015 to 2023) (Table 1).

## Preprocedural Risk Factors

Preprocedural risk factors include age, history of CKD, diabetes mellitus, heart failure, atherosclerotic burden, fluid status, nephrotoxic agents, and global surgical risk [7]. Despite exclusion of patients with ESRD from pivotal TAVR trials, data shows that over 50% of high-risk patients suffer > 3a CKD and about 10% of them suffer > 4 CKD, with the risk of AKI increasing significantly at each stage of CKD advancement [10]. Of 7112 cases performed between 2016 and 2019, AKI occurred in 629 (8.8%). In-hospital AKI was significantly associated with the risk of 1-year mortality. Additionally, by evaluating the ratio of contrast volume to baseline estimated glomerular filtration rate (CV/eGFR), CV/eGFR > 2 and CV/eGFR > 3 were associated with AKI. There was also a correlation between the use of general anesthesia for TAVR and the risk of developing AKI [11]. While advanced imaging with fluoroscopy fusion images and TEE could potentially make contrast-free TAVR feasible, trends have shifted away from general anesthesia, thus limiting the use of procedural TEE in some settings [12]. Patients who present for TAVR with known reduced renal function are at great risk given the strong predictor of long-term mortality post-TAVR. Additional studies have evaluated and found that existing atherosclerosis, especially renal artery stenosis, was a predictor of AKI in 19% who did develop AKI; although these studies have shown this association, larger population validation is needed. Furthermore, risk factors such as heart failure and peripheral vascular disease are independent risk factors associated with AKI post-TAVR [13•].

## Intraprocedural Risk Factors

Intraprocedural risk factors include approach route, moderate paravalvular aortic regurgitation, renal embolization, rapid ventricular pacing, exposure to contrast media, bleeding complications, duration of hemodynamic instability, hypotension, blood transfusion, or conversion to open surgery [14]. Study has shown that patients who underwent TAVR via transfemoral (TF) approach compared to those who had transapical (TA) or transaortic (TaO) approach had significantly less AKI post-TAVR. Another meta-analysis further supported this finding, showing across thirteen studies, the risk of AKI is 57% less likely to occur in TF-TAVR approach compared with non-TF approaches [15]. This highlights that non-TF approaches are more invasive, more time consuming, and contribute to intraoperative hemodynamic instability that negatively impacts renal function and can ultimately lead to post-TAVR AKI. This is also compounded by general findings that non-TF approaches are generally at higher risk for blood transfusion that may help explain the multifocal etiology regarding increased AKI risk post-TAVR. Lastly, TA and TaO approaches generally require general anesthesia and as opposed to MAC, which could further contribute to hemodynamic instability and increase duration of the procedure. Patients undergoing general anesthesia compared to monitored anesthetic care have shown no significant difference in 30 day mortality in some meta-analyses but there is some evidence in smaller studies, showing that general anesthesia as an independent risk factor for the development of post-TAVR AKI [16]. This can potentially result in an increase of the patient’s risk of post-TAVR AKI; however, further studies are needed to stratify general anesthesia as an independent risk factor for post-TAVR AKI.

## Postprocedural Risk Factors

Postprocedural risk factors include hemodynamic instability, postoperative bleeding, postoperative aortic insufficiency (AI) and/or paravalvular leak, anemia, and decreased left ventricular ejection fraction. While it is difficult to find existing literature to explicitly categorize postoperative blood transfusions, in patients who developed post-TAVR AKI, 55% of them had received *packed red blood cells* (pRBCs) transfusion, while only 21% of the patients who did not receive pRBCs develop post-TAVR AKI (*p* = 0.002) [17•]. To address hemodynamic instability, it is well understood that renal perfusion is highly dependent on cardiac output, and therefore, it is reasonable to deduce that prolonged postoperative hemodynamic instability can result in post-TAVR AKI. It is not well appreciated in the current literature the degree to which post-TAVR hypotension, vasopressors, and inotropes directly contribute to the pathogenesis of post-TAVR AKI. Thus, further prospective studies are warranted to further explore these findings. Highlighting other postprocedural risk factors, one study noted that postoperative inotropes and vasopressors use was independently associated with the development of post-TAVR AKI, with the caveat that concurrent intraoperative inotropes and vasopressors administered were not associated with the development of post-TAVR AKI. These findings suggest further prospective studies are needed to further stratify the degree of impact inotropes and vasopressors on post-TAVR AKI in the perioperative period. Post-TAVR AKI has been shown in multiple studies that it is an independent risk factor for increased short-term and long-term mortality [18]. Multiple factors must be accounted for when evaluating a patient’s total risk assessment in the setting of known or precipitated AKI in the perioperative period. In addition, it must be recognized that while no intervention is without risks, risk factors can be stratified into modifiable and non-modifiable risk factors (Table 2).

## Perioperative Management

### Pre-TAVR

AKI reduction strategies vary based on preoperative risk factors and clinical assessment at the time of the procedure. Current strategies pre-TAVR include ensuring appropriate fluid volume status at the time of the procedure. This is especially critical in the setting of known CKD where appropriate diuresis must be done prior to proceeding with TAVR. Reducing IV contrast exposure such as spacing the interval between CT angiography, cardiac catheterization, and TAVR may be another strategy for post-TAVR AKI risk reduction; however, it is unclear if contrast media exposure to what degree if any is an independent risk factor for post-TAVR AKI and continues to be studied [19]. During TAVR, use of contrast-sparing techniques should be implemented, as well as adopting transradial approach for PCI prior to TAVR and TF approach as mentioned previously if the patient’s vessel anatomy allows for it. Strong communication between anesthesiologist and cardiologist performing the procedure to avoid intraoperative hypotension, as well as shortening periods of rapid pacing runs and inotropic and vasoactive agents’ usage, is also critical. Lastly, it may be beneficial to establish institutional policies that ensure conservative blood transfusion thresholds, especially in the setting of known CKD patients, to further assist with post-TAVR AKI prevention.

### During TAVR

Patients who receive TAVRs generally follow the minimalist TAVR care pathway, which serves to decrease complications. One such example would be avoiding general anesthesia when possible. Doing so, the team can help prevent significant intraoperative hemodynamic instability, decreased vascular tone and afterload, atelectasis, and postoperative cognitive dysfunction, all of which can prolong length of stay and potentially contribute to overall morbidity and mortality.

### Post-TAVR

Postoperative management is equally critical to ensuring the integrity of the procedure as well as recognizing any potential new complications in the postoperative period prior to patient’s discharge. Post-TAVR fluid management has been shown to be protective as long as it is deemed appropriate for the specific TAVR patient. Specifically, fluid prophylaxis, when indicated, should involve isotonic fluids for volume expansion with normal saline as the preferred method [20]. In general, prophylaxis is indicated for patients who already have AKI, or an eGFR less than 30 ml/min/1.73^2^, and are not undergoing maintenance dialysis [21]. However, many other factors must also be considered, including presence of heart failure, or other hypervolemic conditions. Although there are not specific guidelines for timing, volume, and rate of volume expansion, there is some evidence showing longer regimens (12 h) having conferred lower risk of AKI compared with shorter regimens [22, 23].

## Conclusions

Post-TAVR AKI can be mitigated when the TF-TAVR approach is implemented compared to non-TF approaches. Additionally, patients with preexisting CKD have markedly increased morbidity and mortality when undergoing TAVR. Moreover, patients who require blood products intraoperatively in the setting of hemodynamic instability potentiates post-TAVR AKI risk. Currently, there is no consensus on fluid management in the perioperative setting; thus, future studies are warranted to help provide further guidance. Given that TAVR is continuously expanding its eligible patient population, opportunities remain to continue to optimize and potentially standardize delivery of care to further prevent post-TAVR AKI and ultimately improve periprocedural outcomes.

## Figures and Tables

**Table 1:** Major studies of post-TAVR AKI from 2015 to 2023

Study	Design	Outcomes
Oguri A. 2015	Retrospective cohort study (*n*=2929), France2 registry	CKD 3b, 4, and 5 correlate with poor outcome and are considered a significant risk for TAVI
Merchant, A.M. 2019	Retrospective cohort study, single-center study (*n*=116)	Transfusion of pRBCs but not nadir perioperative Hgb independently associated with post-TAVR AKI (OR=1.67 per unit, 95% CI 1.13–2.47; *p=* 0.01)
Crimi G. 2022	Prospective cohort study, multi-center study (*n*=2696)	Both AKI and CKD significantly and independently affected primary outcome (HR, 3.06 (95%CI, 2.01–4.64], *p*<0.001 and HR, 1.82 [95% CI 1.27, 2.65], *p*<0.01, respectively)
Alzu’bi H. 2022	Systematic review and meta-analysis (36 studies, *n*=70.406)	AKI was documented in 4857 out of 50,395 (9.6%) patients that underwent TF TAVR compared to 3155 out of 19,721 (16%) patients who underwent TA-TAVR, with a risk ratio of 0.49 (95% CI, 0.36–0.66; *p*<0.00001)
Wang, Jiayang. 2017	Systematic review and meta-analysis (*n* = 13 studies)	A total of 9 factors were associated significantly with an increased risk for post-TAVI AKI, including New York Heart Association (NYHA) class IV (OR 7.77,95% CI 3.81–15.85; *p*<0.0001), previous CKD (OR 2.81; 95% CI 1.96–4.03, *p* <0.0001), requirement of red blood cell transfusion (OR 203.95% CI 1.59–2.59, *p*<0.0001), previous peripheral vascular disease (PVD) (OR 1.72, 95% CI 1.39–2.14, *p*<0.0001), previous hypertension (OR 1.66, 95% CI 1.20–2.31, *p*=0.002), previous atrial fibrillation (OR 1.63, 95% CI 1.11–2.39, *p=* 0.012), previous congestive heart failure (OR 1.51, 95% CI 1.09–2.10, *p*=0.013), previous diabetes mellitus (OR 1.34, 95% CI 1.11–1.61,*p*=0.002), and previous stroke (OR 1.29, 95% CI 1.02–1.62, *p*=0.032)

*AKI*, acute kidney injury; *CI*, confidence interval; *CKD*, chronic kidney disease; *HR*, hazard ratio; *NYHA*, New York Heart Association; *OR*, odds ratio; *PVD*, peripheral vascular disease; *TA*, trans-apical; *TF*, trans-femoral; *TAVR/TAVI*, transcatheter aortic valve replacement/implantation

**Table 2:** Modifiable and non-modifiable risk factors leading to AKI

	Modifiable risk factors	Non-modifiable risk factors
1	Access method	Preexisting heart failure
2	Perioperative hypotension events	Peripheral artery disease
3	Preoperative anemia needed blood transfusion	Diabetes mellitus
4	Rapid pacing during balloon-expandable valve deployment	Aortic atheroma burden
5	Intraoperative bleeding causes hemodynamic changes	Chronic kidney disease

*AKI*, acute kidney injury
